# The future prospects of Lithuanian family physicians: a 10-year forecasting study

**DOI:** 10.1186/1471-2296-6-41

**Published:** 2005-10-04

**Authors:** Liudvika Starkiene, Kastytis Smigelskas, Zilvinas Padaiga, Jack Reamy

**Affiliations:** 1 Department of Preventive Medicine, Kaunas University of Medicine, Mickeviciaus str. 9, LT-44307 Kaunas, Lithuania; 2 Graduate Program in Health Services Administration, Xavier University, Ohio, USA

## Abstract

**Background:**

When health care reform was started in 1991, the physician workforce in Lithuania was dominated by specialists, and the specialty of family physician (FP) did not exist at all. During fifteen years of Lithuania's independence this specialty evolved rapidly and over 1,900 FPs were trained or retrained. Since 2003, the Lithuanian health care sector has undergone restructuring to optimize the network of health care institutions as well as the delivery of services; specific attention has been paid to the development of services provided by FPs, with more health care services shifted from the hospital level to the primary health care level. In this paper we analyze if an adequate workforce of FPs will be available in the future to take over new emerging tasks.

**Methods:**

A computer spreadsheet simulation model was used to project the supply of FPs in 2006–2015. The supply was projected according to three scenarios, which took into account different rates of retirement, migration and drop out from training. In addition different population projections and enrolment numbers in residency programs were also considered. Three requirement scenarios were made using different approaches. In the first scenario we used the requirement estimated by a panel of experts using the Delphi technique. The second scenario was based on the number of visits to FPs in 2003 and took into account the goal to increase the number of visits. The third scenario was based on the determination that one FP should serve no more than 2,000 inhabitants. The three scenarios for the projection of supply were compared with the three requirement scenarios.

**Results:**

The supply of family physicians will be higher in 2015 compared to 2005 according to all projection scenarios. The largest differences in the supply scenarios were caused by different migration rates, enrolment numbers to training programs and the retirement age. The second supply scenario, which took into account 1.1% annual migration rate, stable enrolment to residency programs and later retirement, appears to be the most probable. The first requirement scenario, which was based on the opinion of well-informed key experts in the field, appears to be the best reflection of FP requirements; however none of the supply scenarios considered would satisfy these requirements.

**Conclusion:**

Despite the rapid expansion of the FP workforce during the last fifteen years, ten-year forecasts of supply and requirement indicate that the number of FPs in 2015 will not be sufficient. The annual enrolment in residency training programs should be increased by at least 20% for the next three years. Accurate year-by-year monitoring of the workforce is crucial in order to prevent future shortages and to maintain the desired family physician workforce.

## Background

Lithuania is situated in the Eastern Europe and is the biggest of three Baltic States, with an area of 65,000 square kilometres. Its population of 3.4 million is predominately Lithuanian with the ethnic minorities of which 6.7% are Polish, 6.3% are Russians, and 1.2% are Byelorussians. Lithuania was a part of Soviet Union until it declared its independence in 1990 [1].

The start of health care reform in 1991 found the supply of physician human resources in Lithuania dominated by specialists, and the specialty of family physician (FP) did not exist. The Soviet model of health care, which existed in Lithuania until independence, was based exclusively on the exaggerated focus and development of the hospital level, whereas the need to develop the primary health care was ignored [2].

The main goal of the health reform – to shift health care services to primary health care level – was severely hindered by the absence of FPs to provide these services. At the beginning of the reform effort, the specialty of FP was not popular with physicians as it was considered to be non-prestigious, and the system of reimbursement of FPs was inefficient [3]. In 1992, retraining courses for practicing district physicians and paediatricians to become family physicians were launched. However, these courses had limited success due to the lack of teachers in the field. Significant changes in the system started in 1994, when regulations for FPs' training in residencies were adopted. In parallel with regular residency programs, interruptive residency programs for retraining physicians to become FPs were started at Kaunas University of Medicine and Vilnius University. The aim of interruptive residency programs was to retrain practicing district physicians and paediatricians into FPs, using well-structured 10-month program. It was broken down into blocks of 2 weeks to ensure that physicians did not have to leave their jobs for a long period of time. During the period of 1994–2003, 1,908 FPs were trained, of which 77% were trained in the interruptive residencies or retraining courses. Enrolment in regular training programs was approximately 35 medical residents in 2000–2004; in addition, approximately 66 medical residents were annually admitted to training in interruptive residency programs [4]. However, in 2004, admission to interruptive residency was stopped, as it was decided that the sufficient number of FPs have been trained.

Even though the number of FPs has increased very rapidly during the last fifteen years reaching 48.6 per 100,000 population in the beginning of 2005, FPs as percentage of all physicians was only about 12%. In the other countries of the European Union, this percentage was much higher (with exception of Latvia where it was 15%) – the United Kingdom and Germany were around 30%, and the average of 25 countries of the European Union was 23%. Interestingly enough, Lithuania has one of the highest physician to 100,000 population ratios in the EU, while having one of the lowest FPs to 100,000 population ratios (Table 1) [5,6].

**Table 1:** Number of FPs and physicians per 100,000 population in 1997 and 2003

Country	FPs per 100,000 population		Physicians per 100,000 population	
	
	1997	2003*	1997	2003*
Lithuania	7.0	48.6	414.3	391.1
Latvia	16.1	45.2	296.0	298.5
Estonia	52.3	62.9	323.9	314.1
Germany	109.6	104.3	312.8	336.9
United Kingdom	60.4	62.8	188.6	212.6
EU-25	56.2	64.1	271.9	278.4

* Or the latest available year

The demographic characteristics of Lithuanian FPs are very similar to those of the overall physician population, except for the age structure. While 19.8% of the physicians were older than 60 years; only 2.5% of FPs were older than 60 years, and only 4.9% older than 50 years [7]. FPs are unequally distributed in ten counties of Lithuania, with the highest FP to population ratios being in the cities and the lowest ratios in rural areas. The physician workforce in Lithuania has been traditionally dominated by women (70%); in family medicine that proportion is even higher (84.9%) [5,7].

Since 2003, the Lithuanian health care sector has undergone restructuring in order to optimize the network of health care institutions as well as the delivery of services; specific attention has been paid to the development of services provided by FPs and shifting more health care services from the hospital level to primary health care level. According to current legislation, one FP should serve not more than 2,000 inhabitants [8], however in 2005 one FP served on average 2,058 inhabitants. As defined in the Restructuring Strategy of Health Care Institutions, the number of visits to FPs should increase by 18.7% by the end of 2005 compared with 2003. In order to meet the goals of the strategy, a sufficient number of FPs will need to be available [9].

In this paper we project the supply of and the requirement for FPs in Lithuania until 2015. Here we provide ten-year planning projections, which are essential if an adequate workforce of FPs is to be available to meet future needs. Correspondingly, changes in the number of medical residents enrolled in training programs should be managed to meet the requirements of this plan.

## Methods

The standard approach to planning of health human resources was used in this study. It included the projection of supply, the projection of requirements and an analysis of the gap between supply and requirement [10,11].

### Supply projections

A computer spreadsheet simulation model was used to project FP supply from 2006 to 2015 [12]. Three supply projection scenarios were used for planning purposes (Table 2). The supply of active FPs on January 1, 2005, the projected Lithuanian population by 2015, losses from the profession (due to death, retirement and migration), entry into profession from residency programs (adjusted for the drop out rate), and the duration of residency studies in family medicine (3 years) were used in this model.

**Table 2:** Supply projection scenarios and assumptions

Variables	First scenario	Second scenario	Third scenario
Duration of residency studies	3 years	3 years	3 years
Population projections	Optimistic	Medium	Pessimistic
Annual mortality rate	0.47%	0.47%	0.47%
Annual retirement rate	At the age of 71 years	At the age of 71 years	At the age of 66 years
Annual migration rate	Optimistic rate	Medium rate	Pessimistic rate
Enrolment in residency studies	Increased by 20% (46)	At the level of 2004 (38)	Decreased by 20% (30)
Drop out rate during residency	1%	1.5%	2%

#### Population projections

Projected Lithuanian population by 2015 according to three possible scenarios: medium, optimistic and pessimistic was obtained from the Department of Statistics [13].

#### Annual mortality rate

There was no accessible data on the annual mortality rate of FPs, therefore we used a weighted average of the age-specific (25–64 years) and the gender-specific (84.9% women and 15.1% men) mortality rates of the general Lithuanian population obtained from the Department of Statistics [1]. This assumption tends to underestimate the annual mortality rate, since according to other studies the physician mortality rate is usually somewhat higher [10,14].

#### Annual retirement rate

Since no reliable data were available on the average annual retirement rate of Lithuanian FPs, we applied the method by Pace et al in order to calculate it [15]. We used the data from Physician License Registry [7]. The retirement age was set to be 66 years and then assumption was made that one fifteenth of the group of FPs aged more than 50 years would retire annually. In the other scenario the retirement age was set to be 71 years and then assumption was made that one fifteenth of the group of FPs aged more than 55 years would retire annually.

#### Annual migration rate

Source data for the annual drop out from profession due to migration was obtained from the Ministry of Health (data for the period of May 1, 2004 – April 30, 2005). The pessimistic rate was calculated using the following data: dividing the number of FPs who emigrated during the first year by the total number of FPs in 2005, and multiplying by 100 in order to get the annual migration rate. This rate was taken as the worst estimate because it was unlikely that the migration rate, which was observed 12 months after joining the European Union, would remain at the same level for the period of ten years. Medium rate was assumed to be half of the pessimistic rate, and optimistic rate – half of the medium rate.

#### Annual enrolment in residency programs and drop out rate

Numbers of annual medical resident enrolment in FPs' training programs in 2000–2004 as well as the number of graduates in 2000–2004 were obtained from the Ministry of Health. Enrolment numbers in the residency programs starting with 2005 were left at the level of 2004, increased or decreased (depending on the scenario) and then converted into future annual number of graduates using three different drop out rates (1%, 1.5% and 2%) established by Lovkyte [14].

### Requirement projections

Requirement of FPs until 2015 was estimated using three different approaches:

1) The first approach was based on the survey conducted in 2000 by use of the Delphi survey technique. To determine the goal of FP workforce planning that should be reached by 2015, we surveyed the deans of the Faculties of Medicine, members of the National Board of Health, county chief physicians, directors of the Territorial Sickness Funds and the State Sickness Fund, and representatives of the Ministry of Health and the WHO Liaison Office. Out of a total of 34 questionnaires sent out, 23 were completed and returned in the first round. In the second round, the questionnaires were sent only to the 23 respondents of the first round, of whom 15 responded [2].

2) The second approach was based on the number of visits to FPs in 2003 adjusted by growth of these visits (by 18.7% until the end of 2005), defined in the Restructuring Strategy of Health Care Institutions [9]. Unfortunately, further goal on estimated growth is not available; therefore we assumed that there will be no further growth in number of visits to FPs in 2006–2015. Breakdown of health care services provided in 2003 by patients' age groups and gender was multiplied by projected changes in the population. Afterwards the projected number of visits was increased by 18.7% and converted into a number of FPs, using the number of visits to one FP in 2003.

3) The third scenario was based on the provision by the Ministry of Health that one FP should serve no more than 2,000 inhabitants [8].

### Gap analysis

A gap analysis was performed comparing supply and requirement projections in order to identify future shortages or surpluses of FPs. We also attempted to determine the factors that had the largest impact on future FP workforce.

## Results

### Supply projections

Prior to projection exercise we had to make some calculations regarding the mortality, the retirement and the migration of FPs.

#### Population projections

Lithuania is characterized by a declining and aging population. It is expected that by 2015 the number of inhabitants will decrease according to all scenarios: from 100,000 according to the optimistic scenario to 250,000 according to the pessimistic scenario [13].

#### Annual mortality rate

The mortality of women in the 25–64 age group was 0.37% and the mortality of men was 1.06% in 2004. The weighted average of mortality in the 25–64 age group adjusted by gender (84.9% women and 15.1% men) was 0.47% [1].

#### Annual retirement rate

Family practice is a young physician specialty with relatively few physicians being at retirement age [7]. Only four physicians per year could be expected to retire using 71 years as the retirement age (since there were 54 physicians aged 56 years or older, one fifteenth of them should be at retirement age or older). Using a lower retirement age – 66 years, on average 11 physicians per year could be expected to retire (since there were 169 physicians aged 51 years or older, one fifteenth of them should be at retirement age or older).

#### Annual migration rate

The pessimistic annual migration rate was calculated to be 2.2%, using the following reasoning: 36 FPs left the country during 12 months, and there were 1,665 FPs in 2005. Medium rate was calculated to be 1.1% and optimistic rate 0.6%.

#### Annual enrolment in residency programs and drop out rate

Different annual enrolment rates (30, 38 and 46) also resulted in different gains to profession, varying from 444 to 569.

**Figure 1:**
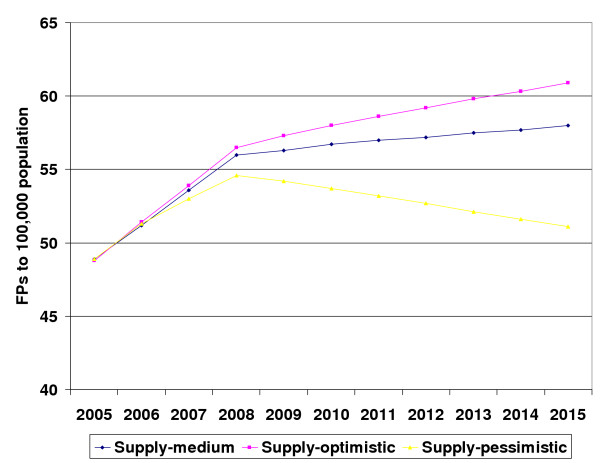
Projections of FPs supply (FPs to 100,000 population ratio) according to the three scenarios.

Figure 1 summarizes the projections of FPs' supply according to three scenarios. According to the first scenario, FPs-to-population ratio would be higher by 24.6% in 2015 than it was in 2005. The second scenario forecasts increase by 18.6%. The third projection also indicated increase by 4.5%, which would result in the ratio of 51.1 per 100,000 population.

All three supply projections were equally influenced by the annual mortality rate (Table 3). The biggest differences in supply scenarios were caused by different migration rates, enrolment numbers in training programs and the retirement age. If the retirement age was set at 66 years, 110 FPs could be expected to retire during 10-year period, compared with only 40, if retirement was 71 years. If migration remained stable at the current rate, 381 FPs could be expected to leave Lithuania in 2006–2015. The rise in the supply curves until 2008 was mainly caused by the higher annual number of graduates than in the consecutive years (Figure 1.). In addition to the graduates of regular residency programs (30–46 depending on scenario), it included graduates of interruptive residency programs (around 65 each year). Later fall was related to discontinued admission to these programs since 2004. The drop out rate from training programs is also reflected in the number of graduates, but due to very small numbers it had little influence on the future supply of FPs.

**Table 3:** Variables / assumptions and their influence on estimated losses / gains to the profession of FPs in 2006–2015

Variable	Assumption	Estimated losses from profession during 2006–2015	Estimated gains to profession during 2006–2015
Annual mortality rate	0.47%	88	-
Annual retirement rate	At the age 71 years	40	-
	At the age of 66 years	110	-
Annual migration rate	0.6%	116	-
	1.1%	207	-
	2.2%	381	-
Enrolment in residency programs	Decreased by 20%	-	444
	At the level of 2004	-	503
	Increased by 20%	-	569
Drop out rate during residency	1%	5	-
	1.5%	6	-
	2%	6	-

### Requirement projections

According to the first scenario, the requirement for FPs was 67.0 per 100,000 population [2]. This scenario would also have one FP serving 1,500 inhabitants.

The second scenario was based on the number of visits and their growth (Table 4). In 2003, there were 1,500 family physicians, who were visited more than 5.4 million times. Table 4 indicates the breakdown of population and visits by gender and age groups in 2003 and 2015. Lithuania is characterized by a declining and aging population. It is expected that by 2015 the number of inhabitants will decrease on average by 185 thousand; almost 100 thousand of them will be in the age group under 18 years. Logically, the number of visits should also decrease and if the goal to increase the number of visits by 18.7% until the end of 2005, as defined in the Restructuring Strategy of Health Care Institutions, was not taken into account, the requirement for FPs was 44.0 per 100,000. However, taking into account increasing number of visits, 52.2 FPs per 100,000 population would be needed to ensure the proper provision of services.

**Table 4:** Population and number of visits to FPs in 2003 and 2015

Gender, age group	Population in 2003 (in thousands)	Number of visits in 2003 (in thousands)	Projected population in 2015 (in thousands)	Projected number of visits in 2015 (in thousands)	Projected number of visits in 2015, adjusted with 18.7% increase (in thousands)
Males:					
0–18	438.5	736.3	336.5	565.0	670.7
19–44	650.5	537.6	597.1	493.5	585.8
45–64	353.1	496.1	409.5	575.5	683.0
>65	175.2	401.2	183.4	419.8	498.4
Totally, males	1617.3	2171.2	1526.5	2053.8	2437.9
Females:					
0–18	418.8	720.9	320.0	550.0	653.9
19–44	660.5	734.6	602.5	670.0	795.3
45–64	431.5	863.2	473.1	946.5	1123.6
>65	334.5	933.3	355.3	991.5	1176.9
Totally, females	1845.3	3252.0	1750.9	3158.0	3749.7
Totally	3462.6	5423.2	3277.4	5212.8	6187.6

According to the third scenario, the requirement was based on the ruling by the Ministry of Health that one FP should serve not more than 2,000 inhabitants, i.e. 50.0 FPs per 100,000 population would be required in 2015.

### Analysis of a gap between supply and requirement

In our last step we compared the three supply projections with three requirement projections. As shown in Table 5, the third requirement scenario would be exceeded by all three supply scenarios. The requirement indicated by the second scenario would be exceeded by the supply projected according to the first and the second scenarios. None of the supply scenarios would reach the requirement indicated by the first scenario (67.0); even the supply according to the first scenario would be lower (60.9 FPs per 100,000).

**Table 5:** Gap between supply and requirement projections (FPs per 100,000 population)

Scenarios		Requirement scenarios for FPs per 100,000 population in 2015		
		
		First (67.0)	Second (52.2)	Third (50.0)
Supply scenarios of FPs per 100,000 population in 2015	First (60.9)	-6.1	8.7	10.9
	Second (58.0)	-9.0	5.8	8.0
	Third (51.1)	-15.9	-1.1	1.1

*NB. Minus indicates that the requirement will be higher than the supply*

## Discussion

Although recognized as an important part of health care system reform, the comprehensive planning of an FP workforce has not been a high priority in Lithuania over the last fifteen years. The national policy has been mostly limited to establishing training and retraining programs. This study is the first attempt to provide ten-year planning projections essential to ensure an adequate FP workforce in the future.

As mentioned earlier in this article, Lithuania started with no family physicians after restoration of independence. In 1996 an international expert group led by Corder set a target to increase the percentage of FPs to 20% of the overall physician population by 2005 [16]; however the goal was not reached and currently FPs make up only 12% [5]. Another target to train 2,400 FPs by the year 2010 was set in the primary health care development program, adopted by the Ministry of Health in 2000 [17]. If enrolment in residency programs was increased by 20% (as indicated by the first supply scenario), likely this training target could be reached; nevertheless the number of practicing FPs might be not sufficient. For example, out of 1,908 FPs who graduated through 2004, 91.2% held a license, but only 78.6% were practicing [5,7].

While family medicine in Lithuania has been dominated by women (84.9%), unlike other countries, women do not tend to work part-time or see fewer patients than male physicians, mainly due to an unfavourable payment system [7]. Maternal leave is also basically limited to one year due to financial disincentives to prolong it. The gender composition of medical school graduates has remained quite steady over the last decade, and it is unlikely that the number of women FPs will change in the future [2].

The migration of FPs should be monitored with particular concern. According to a survey of Lithuanian physicians conducted in 2004, 26.8% intended to leave the country and 3.8% have made a definite decision to do so. Younger age was a risk factor for leaving and is particularly important in case of FPs, since the vast majority of them are of young age. As the main reasons for leaving, salary and professional career differences were identified, and it is unlikely that this gap between Lithuania and old European Union members will diminish in a few years [18]. It is realistic that 1.1% of FPs could emigrate annually. The emigration rate of 2.2% is unlikely, because it assumes that the emigration rate, which was observed during the 12 months after accession to the European Union, would be sustained for the ten-year period.

Due to social uncertainties and an unfavourable retirement policy Lithuanian physicians are reluctant to retire at an earlier age; according to Lovkyte, 45.7% of all Lithuanian physicians were still practicing at the age of 66 and later, and it is unlikely that all would retire at once [14].

The number of visits to family physicians increased almost two-fold from 3 million in 2001, the first year for which data on the number of services provided is available, to 5.4 million in 2003. During the same period the number of visits to specialist physicians working in the primary health care setting decreased from 4.8 million in 2001 to 4.2 million in 2003 [19]. FPs currently have very busy practices, allocating an average of 10 minutes to each patient. Recent studies indicated that total job satisfaction of family physicians in Lithuania was relatively low. Compensation, high job demands, social status, and high patient load were among the key factors that caused their dissatisfaction and were significant predictors of psychosocial stress. Unfavourable job environment can also reduce the attractiveness of the profession and result in talented medical graduates choosing other medical or non-medical specialties [20,21].

In our opinion, the second supply scenario, which takes into account a 1.1% annual migration rate, a stable enrolment to residency programs and a later retirement, is the most probable. The first requirement scenario is the best as it was based on the opinion of well-informed key experts in the field. These experts took into account not only the historic number of visits, the short-term changes in the number of visits or minimal needs of the population, but also complex factors having an impact on this profession as well as future changes in health care system.

Several recommendations could be suggested, however some of them would be difficult to implement. As a general course of action, majority of the planning organizations favour adjustments to enrolment to training programs as the best long-term solution to any anticipated imbalances between expected supply and estimated requirement. This study is not exceptional in this sense and a recommendation is made to increase the enrolment to FPs' training program by 20% at least for three years in order to prevent future shortages of FPs. In the future, projections should be updated and further recommendations should be drawn. Another recommendation would be to increase retention rates in the profession, via implementing reformed and significantly improved financial and non-financial incentive system (the examples would include increased per capita reimbursement for FPs mixed with fee-for-service payments, better working conditions, lower patient load, improved access to continuous medical education courses, etc.). This could also contribute to lower emigration rates. Other recommendations such as assigning more duties to professional nurses, who would be trained to undertake part of family physicians' duties, go beyond the scope of this study.

## Conclusion

Family medicine in Lithuania will face several challenges in coming years. There will likely be a lack of approximately 9 FPs per 100,000 (or 300 FPs for the whole population) in 2015, which should be considered as increased duties and responsibilities are assigned to them. Job satisfaction of FPs is relatively low, with compensation, high job demands, social status, and high patient load as key factors in causing dissatisfaction and psychosocial stress.

We recommend that the enrolment in residency programs be increased by 20% at least for the next three years. Special attention should be paid to monitoring of retirement and retention rates in profession. Every fifth graduate was not practicing in Lithuania, as he / she either chose a better paid job or moved to another country. A better retention program would reduce training requirements to achieve the desired workforce supply. Achieving a balance between the supply and the requirements is very complex, but important task in order to ensure the appropriate and efficient functioning of the health care system in the future. Requirement and supply projections should continue to be monitored annually, and be amended, if new trends in any of the FP characteristics emerge or projection assumptions change. Without some more comprehensive registry or means to link the existing databases, complete information on the FP workforce in Lithuania will remain difficult if not impossible to obtain.

## Competing interests

The author(s) declare that they have no competing interests.

## Authors' contributions

All authors participated in designing the study, making data analysis, writing the original text and read and approved the final manuscript.

## Pre-publication history

The pre-publication history for this paper can be accessed here:



## References

[B1] Department of Statistics of the Republic of Lithuania (2004). Demographic yearbook 2003 Vilnius.

[B2] Lovkyte L, Reamy J, Padaiga Z (2003). Physicians Resources in Lithuania: Change Comes Slowly. Croat Med J.

[B3] Valius L, Grabauskas V (2000). Primary health care in Lithuania: legal basis, process of reform and problems. Health Policy in Lithuania in the 21st century Proceedings of the third National Conference on Health Policy in Lithuania.

[B4] Ministry of Health of Republic of Lithuania (2004). Training of family physicians in 1994–2003 Vilnius.

[B5] Lithuanian Health Information Center (2003). Population health and activities of health care facilities in Lithuania Vilnius.

[B6] World Health Organization Regional Office for Europe (2005). European health for all database (HFA-DB).

[B7] Ministry of Health of Republic of Lithuania (2004). Physician License Registry data for January 1, 2004. Report No: 2004-03-18-48/8 Vilnius.

[B8] Ministry of Health of the Republic of Lithuania (2000). On ratification of service list, basic prices, organization and payment for primary health care services. Valstybes zinios.

[B9] Ministry of Health of the Republic of Lithuania (2004). On ratification of restructuring plans of counties' health care institutions. Valstybes zinios.

[B10] Australian Medical Workforce Advisory Committee (2000). The general practice workforce in Australia: supply and requirements 1999–2010. AMWAC Report 20002 Sydney.

[B11] Australian Medical Workforce Advisory Committee (2000). Medical workforce planning in Australia. Aust Health Rev.

[B12] Dewdney J (2000). WPRO/RTC health workforce planning workbook.

[B13] Department of Statistics of the Republic of Lithuania (2004). Prognoses of Lithuanian population 2005–2030 Vilnius.

[B14] Lovkyte L (2004). Planning projections of physician supply and requirement in Lithuania until 2015: doctoral dissertation.

[B15] Pace KT, Provan JL, Jewett MA (1999). The urology workforce in Ontario for the 21st century: feast or famine?. Can J Surg.

[B16] Corder DW (1996). Planning of physician supply in Lithuania. Report to the Ministry of Health of Republic of Lithuania Vilnius.

[B17] Ministry of Health of the Republic of Lithuania (2003). On adoption of primary health care development program. Order of the Minister of Health No 441 Vilnius.

[B18] Stankunas M, Lovkyte L, Padaiga Z (2004). The survey of Lithuanian physicians and medical residents regarding possible migration to the European Union. Medicina (Kaunas).

[B19] Kaunas University of Medicine (2004). Strategic planning of health human resources in Lithuania in 2003–2020. Annual report to the Ministry of Health Kaunas.

[B20] Buciuniene I, Blazeviciene A, Bliudziute E (2005). Health care reform and job satisfaction of primary health care physicians in Lithuania. BMC Family Practice.

[B21] Vanagas G, Bihari-Axelsson S (2005). The factors associated to psychosocial stress among general practitioners in Lithuania. Cross-sectional study. BMC Health Services Research.

